# Brachytherapy Versus Stereotactic Body Radiotherapy for Cervical Cancer Boost: A Dosimetric Comparison

**DOI:** 10.7759/cureus.37235

**Published:** 2023-04-07

**Authors:** Zineb Dahbi, Kouhen Fadila, Vincent Vinh-Hung

**Affiliations:** 1 Radiotherapy, International University Hospital Cheikh Khalifa, Mohammed VI University of Health Sciences (UM6SS), Casablanca, MAR; 2 Medicine, Mohammed VI Polytechnic University, Benguerir, MAR; 3 Radiation Oncology, International University Hospital Cheikh Khalifa, Mohammed VI University of Health Sciences (UM6SS), Casablanca, MAR; 4 Radiation Oncology, University Hospital of Martinique, Fort-de-France, MTQ

**Keywords:** cervical cancer traitments, dosimetry, stereotactic body radiotherapy, brachytherapy, cervical cancer boost

## Abstract

Background: The standard treatment for locally advanced cervical cancer involves chemo-radiation followed by brachytherapy. However, some patients are unable to undergo brachytherapy intensification. Recent advancements in radiation technology have provided several techniques, with stereotactic body radiation therapy (SBRT) theoretically able to mimic the dose distribution of brachytherapy with a high dose gradient.

Methods: We analyzed 20 high-dose-rate intra-cavity brachytherapy plans for women with cervical cancer and simulated an adjunctive stereotactic radiotherapy plan at the same doses used for brachytherapy (21 Gray [Gy] in three fractions). No planning tumoral volume (PTV) margin was added for SBRT dosimetry. We used the dose constraints for brachytherapy from the EMBRACE trial and the dose constraints for SBRT in three fractions. Dose distribution, maximum dose points on target volumes, bladder, rectum, and dose-volume histograms were compared between the two techniques.

Results: The mean volume of the high-risk clinical tumoral volume (CTV) was 64 cm3, and the mean volume of the intermediate-risk CTV was 93 cm3. The mean minimum dose received by 90% of the high-risk CTV (D90 CTV HR) was 17 Gy for brachytherapy versus 8.3 Gy for SBRT. The average minimum dose received by 90% of the intermediate-risk CTV (D90 CTV IR) was 7.5 Gy for brachytherapy versus 8.9 Gy for SBRT. The mean minimum dose delivered to 2cc of the bladder was 74.6 Gy for brachytherapy versus 84.7 Gy for SBRT. The mean minimum dose delivered to 2cc of the rectum was 71.8 Gy for brachytherapy versus 74.7 Gy for SBRT.

Conclusion: We confirmed the dosimetric superiority of brachytherapy over SBRT in terms of target volume coverage and organ-at-risk sparing. Therefore, pending the results of further clinical studies, no current radiotherapy technique can replace brachytherapy for cervical cancer boost after external radiotherapy.

## Introduction

Cervical cancer is a common type of cancer in women, with approximately 570,000 new cases and 311,000 deaths each year worldwide [1]. The incidence of cervical cancer varies significantly by region and socioeconomic status. However, cervical cancer remains a major public health problem in many developing countries where access to human papilloma virus screening and vaccination is limited.

For cervical cancer, the current standard of care for locally advanced stages consists of concomitant chemotherapy and external pelvic radiotherapy, followed by intra-cavitary brachytherapy [2]. The standard dose of radiation therapy used to treat cervical cancer is usually in the range of 45 to 50 Gray (Gy), given in daily fractions of 1.8 to 2 Gy over a period of six weeks. Brachytherapy boost doses vary depending on the type of brachytherapy used, but are generally between 15 and 30 Gy [3,4]. Overall, the exact dose of radiation therapy will depend on a number of factors, including the stage and size of the tumor, presence of pathologic lymph nodes, as well as the patient's overall health.

However, brachytherapy is not feasible in some patients because of unfavorable anatomy, contraindications to anesthesia, comorbidities, and patient refusal for the procedure, or even lack of accessibility to brachytherapy in some countries. For patients unfit for brachytherapy, noninvasive modalities, such as modulation intensity, or stereotactic body radiotherapy (SBRT) are possible.

From a technical point of view, SBRT is a type of external beam radiation therapy that delivers high doses of radiation precisely to a tumor in a few sessions. It is often used to treat small, hard-to-reach tumors in the lungs, liver and spine. Brachytherapy, on the other hand, is a type of internal radiation therapy that involves placing a radioactive source directly into or near a tumor. This can be achieved through implants or by inserting a thin tube into the tumor. Brachytherapy is often used to treat prostate and cervical cancer, as well as some types of head and neck and breast cancer.

Theoretically, SBRT is the most likely substitute for a brachytherapy dose distribution with a sharp dose gradient. Therefore, we aimed to perform a dosimetric comparison between target volume coverage and organ risk sparing, between SBRT and brachytherapy plans, as modalities for cervical boost in locally advanced cervical cancer.

## Materials and methods

Twenty patients with histologically proven locally advanced cervical cancer, epidemiologic and tumoural characteristics detailed in Table 1, previously treated with intra-cavitary brachytherapy boost, were treated with a course of chemo-radiotherapy at 46 Gy in 23 fractions of 2 Gy, five fractions per week. All treatments were performed using the conformal technique: four fields of the box technique, in our radiotherapy center.

**Table 1:** Baseline demographics and disease characteristics. FIGO: International Federation of Gynaecology and Obstetrics

Characteristic	N =20 (%)
Median Age (years)	58.2 ± 9.6
Median Tumor Size (cm3)	7.65 ± 6.0
Tumor differentiation	
Well differentiated	6 (30%)
Poorly differentiated	8 (40%)
Undifferentiated	6 (30%)
Histology	
Squamous cell	13 (65%)
Adenocarcinoma	6 (30%)
Other	1 (5%)
FIGO Stage	
I	2 (10%)
II	4 (20%)
III	10 (50%)
IV	4 (20%)
Median Treatment Duration (days)	64.55 ± 4.3

The brachytherapy was carried out with a Fletcher applicator. A computed tomography (CT) scan with the applicator in place was performed and merged with a magnetic resonance image (MRI) to better delineate high- and intermediate-risk volumes. The target volumes and organs at risk were delineated according to the recommendations of the EMBRACE II protocol [4].

The iso-dose for the brachytherapy plan was copied onto the simulation CT for the external beam radiotherapy, and a dosimetric plan for SBRT was performed on our treatment planning system (ARIA13; Varian Medical Systems, Palo Alto, CA, USA). No planning target volume (PTV) margin was added to the high risk (HR) and intermediate risk (IR) clinical target volume (CTV), i.e. PTV equated CTV. We prescribed three fractions of 7 Gy, administered homogeneously.

We aimed to deliver a target volume as close as possible to the high-risk anatomic CTV, while respecting risk of the dose to other organs. The doses were calculated as equivalent doses delivered in fractions of 2 Gy, such that the doses were equivalent in terms of effectiveness to the prescription dose of 85 Gy at 2 Gy per fraction, assuming an alpha/beta of 10 Gy for target volumes and 3 Gy to pelvic organs at risk. Timmerman dose constraints for three fractions of SBRT were used. The optimization constraints for the SBRT and brachytherapy plans are summarized in Table 2 [5].

**Table 2:** Dose constraints for brachytherapy and stereotactic body radiotherapy plans. SBRT: stereotactic body radiotherapy; EQD2Gy: equivalent dose in 2 Gy fractions

	Rectum	Bladder	Femoral Head	Small Bowel
Constraints for SBRT	V20 Gy < 20 cc D_max_ = 30 Gy	V15 Gy < 15 cc V30 Gy < 5 cc	V22 Gy < 5 cc	V16 Gy < 5 cc V27 Gy < 0.5 cc
Constraints for brachytherapy (EQD2Gy)	D2 cc ≤ 75 Gy	D2 cc ≤ 90 Gy	---	D2 cc ≤ 75 Gy

All plans used the anisotropic analytical algorithm (AAA, version 10.0.28) for dose calculation, with 2.5 mm calculation grid and heterogeneity correction.

Regarding the statistical analysis, all results were compared and analyzed using SPSS software, version 10.0 (IBM Corp., Armonk, NY, USA). A statistical significance level of 0.05 was used (p < 0.05).

The different treatment techniques were applied to the patients’ dataset without any clinical application. This activity obtained the basic ethical approval from the Mohammed VI University of Health Sciences (UM6SS) Ethics Committee (approval SBRT-BT15Juin2020).

## Results

Comprehensive patient demographics are summarized in Table 1; in brief, the median age was 58.2 years and 65% of the patients had squamous cell cervical carcinoma. Half of our patients had stage III disease (International Federation of Gynaecology and Obstetrics [FIGO] 2009). Median tumor size was 7.65 cm. All patients had a first course of conformal external beam pelvic radiotherapy, with concurrent chemotherapy. The median treatment duration was 64.55 days.

Dosimetric characteristics for the target volumes and for the organs at risk for the SBRT plans are included in Table 3. Mean high-risk CTV and intermediate-risk CTV were, respectively, 64 cm3 and 93 cm3. The mean minimal dose received by 90% of the high-risk CTV cases was 17 Gy, versus 8.32 Gy for patients receiving SBRT. The mean minimal dose received by 90% of the intermediate-risk CTV cases was 8.99 Gy, versus 7.5 Gy for patients receiving brachytherapy. The mean near-maximal dose delivered up to 2 cc of bladder was 74.6 Gy for the brachytherapy plan versus 84.7 Gy for the SBRT plan. The mean near-maximal dose delivered up to 2 cc of rectum was 71.8 Gy for the brachytherapy plan versus 74.7 Gy for the SBRT plan.

**Table 3:** Target volumes and organs-at-risk parameters for stereotactic body radiotherapy and brachytherapy plans. SBRT: stereotactic body radiotherapy; EQD2: equivalent dose in 2 Gy fractions; CTV: clinical target volume; HR: high risk; IR: intermediate risk

		SBRT (EQD2)	Brachytherapy	P
CTV HR Alpha/beta =10	Mean Volume (cc)	64		
	Mean D90 (Gy)	8.32	17	0.028*
	Mean D2cc (Gy)	26.24	500	
	Conformity index	0.86	0.76	0.671
CTV IR Alpha/beta =10	Mean Volume (cc)	93		
	Mean D90 (Gy)	8.9	7.5	
	Mean D2cc (Gy)	36.00	84.13	
	Conformity index	0.513	0.842	0.626
Bladder Alpha/beta =3*	Mean D2cc (Gy)	84.7	74.6	0.019*
Rectum Alpha/beta =3*	Mean D2cc (Gy)	74.7	71.8	0.256
Sigmoid Alpha/beta =3*	Mean D2cc (Gy)	75.75	67.9	0.0173*

## Discussion

Stereotactic ablative radiotherapy consists of treating localized tumors by delivering very high doses of focused radiation. This approach has led to an impressive increase in local control in multiple disease sites, including lung, liver, prostate, and spine [6,7]. Theoretically, SBRT dose distributions can mimic the dose fall-off and heterogeneity of brachytherapy plans. SBRT might therefore be useful for patients who are unable to have invasive brachytherapy due to medical comorbidities [8,9].

Historically, multiple attempts to replace brachytherapy with conventionally fractionated radiation therapy have resulted in lower local control rates [10,11]. However, with the advent of highly conformal treatments, such as SBRT, the safety and efficacy of this approach as a boost is being readdressed [12].

For the most part, boost doses range from 15 to 30 Gy for three to five fractions, with mean local control rates of 80% and high-grade toxicity rates of 10% [13,14]. Very few reports have detailed the added margins to PTV volumes. Some have short follow-up periods. Of these, our series had the largest sample size [15].

SBRT can produce a high-quality dose distribution, even for large tumors, but the dose to other organs can be less acceptable than that with brachytherapy plans. For this reason, intra-cavitary brachytherapy (and possibly interstial brachytherapy) is still dosimetrically superior to SBRT in cervical cancer [15].

While in our series, brachytherapy is significantly more effective than SBRT in covering high-risk target volumes and is significantly more protective of the bladder and rectum, a recent review by Albuquerque et al. reported a series of 15 patients undergoing SBRT for cervical cancer boost [16]. Their published dosimetric data matches ours. For instance, the median dose to the rectum was 90.63 Gy with a 2 cc dose of 25.57 Gy, while in our plan, the mean dose to the rectum was 89 Gy, but the authors concluded anyway that for patients who are unable to undergo standard brachytherapy, and thus do not have other treatment options, SBRT is feasible, with the caveat that it would be appropriate only for select patients with modest local control at the cost of possible increased late toxicity [17]. Future studies of SBRT for cervical cancer should be directed at optimizing the volume (possibly gross tumor volume alone), dose, and fractionation for this approach.

Furthermore, a recently published review by Barret et al. considered patients with cervical cancer treated with chemoradiation plus brachytherapy boost, intensity-modulated radiation therapy (IMRT) boost, or SBRT boost. It concluded that there was no significant difference in overall survival (OS) for patients who received SBRT boost versus brachytherapy boost; only IMRT had significantly lower OS than brachytherapy. This provided additional support that SBRT might be an accurate alternative for patients who are unfit for brachytherapy boost [18-20].

The main limitations of this study are the small sample size, the fact that applicators are not intended to be used in SBRT treatments, and the lack of clinical analysis for local control and early-late toxicity data. Further studies with a larger population should be run, in order to assess the potential clinical gain from these radiation techniques for this particular indication.

## Conclusions

For patients who are not eligible for brachytherapy because of particular conditions, other curative treatment options for cervical cancer boost may include external beam radiation therapy with modulation intensity, surgery by radical hysterectomy, or palliative treatments by chemotherapy and immunotherapy. SBRT is a highly precise and targeted form of external radiation therapy that delivers a high dose of radiation to a small specific area in one or more sessions. It can be used as an alternative for cervical cancer patients unfit for brachytherapy boost. Our data confirmed the dosimetric superiority of brachytherapy, compared with SBRT, in terms of target volume coverage and sparing of at-risk organs. Therefore, we look forward to additional clinical trials to assess the outcomes of these approaches.

## References

[1] Sung H, Ferlay J, Siegel RL, Laversanne M, Soerjomataram I, Jemal A, Bray F (2021). Global cancer statistics 2020: GLOBOCAN estimates of incidence and mortality worldwide for 36 cancers in 185 countries. CA Cancer J Clin.

[2] Chino J, Annunziata CM, Beriwal S (2020). Radiation therapy for cervical cancer: executive summary of an ASTRO clinical practice guideline. Pract Radiat Oncol.

[3] Chargari C, Peignaux K, Escande A (2022). Radiotherapy of cervical cancer. Cancer Radiother.

[4] Pötter R, Tanderup K, Kirisits C (2018). The EMBRACE II study: the outcome and prospect of two decades of evolution within the GEC-ESTRO GYN working group and the EMBRACE studies. Clin Transl Radiat Oncol.

[5] Noël G, Antoni D, Barillot I, Chauvet B (2016). [Delineation of organs at risk and dose constraints]. Cancer Radiother.

[6] Gultekin M, Yilmaz MT, Yuce Sari S, Yildiz D, Ozyigit G, Yildiz F (2022). Stereotactic body radiotherapy boost in patients with cervical cancer. J Obstet Gynaecol.

[7] Gao J, Xu B, Lin Y (2022). Stereotactic body radiotherapy boost with the cyberknife for locally advanced cervical cancer: dosimetric analysis and potential clinical benefits. Cancers (Basel).

[8] Lee TH, Song C, Kim IA (2021). Stereotactic ablative body radiotherapy boost for cervical cancer when brachytherapy boost is not feasible. Radiat Oncol.

[9] Chargari C, Renard S, Espenel S (2020). [Can stereotactic body radiotherapy replace brachytherapy for locally advanced cervical cancer? French society for radiation oncology statement]. Cancer Radiother.

[10] Facondo G, Vullo G, DE Sanctis V (2021). Stereotactic body radiation therapy boost in patients with cervical cancer ineligible for brachytherapy. Cancer Diagn Progn.

[11] Otahal B, Dolezel M, Cvek J (2014). Dosimetric comparison of MRI-based HDR brachytherapy and stereotactic radiotherapy in patients with advanced cervical cancer: a virtual brachytherapy study. Rep Pract Oncol Radiother.

[12] Yanez L, Ciudad AM, Mehta MP, Marsiglia H (2018). What is the evidence for the clinical value of SBRT in cancer of the cervix?. Rep Pract Oncol Radiother.

[13] Hennequin C, Dubray B (2013). [Alpha/beta ratio revisited in the era of hypofractionation]. Cancer Radiother.

[14] Gill BS, Lin JF, Krivak TC (2014). National Cancer Data Base analysis of radiation therapy consolidation modality for cervical cancer: the impact of new technological advancements. Int J Radiat Oncol Biol Phys.

[15] Chargari C, Gouy S, Pautier P, Haie-Meder C (2018). [Recent data in cervical cancer: radiation oncologist's perspective]. Cancer Radiother.

[16] Serban M, Kirisits C, Pötter R (2018). Isodose surface volumes in cervix cancer brachytherapy: change of practice from standard (Point A) to individualized image guided adaptive (EMBRACE I) brachytherapy. Radiother Oncol.

[17] Hsieh CH, Tien HJ, Hsiao SM (2013). Stereotactic body radiation therapy via helical tomotherapy to replace brachytherapy for brachytherapy-unsuitable cervical cancer patients - a preliminary result. Onco Targets Ther.

[18] Kubicek GJ, Xue J, Xu Q (2013). Stereotactic body radiotherapy as an alternative to brachytherapy in gynecologic cancer. Biomed Res Int.

[19] Haas JA, Witten MR, Clancey O, Episcopia K, Accordino D, Chalas E (2012). CyberKnife boost for patients with cervical cancer unable to undergo brachytherapy. Front Oncol.

[20] Cengiz M, Dogan A, Ozyigit G (2012). Comparison of intracavitary brachytherapy and stereotactic body radiotherapy dose distribution for cervical cancer. Brachytherapy.

